# Decision making on antenatal screening results: A comparative Q‐method study of women from two Chinese cities

**DOI:** 10.1111/hex.13178

**Published:** 2020-12-14

**Authors:** Dong Dong, Shenaz Ahmed, Elena Nichini, Huso Yi, Hussain Jafri, Yasmin Rashid, Mushtaq Ahmed, Jianfeng Zhu

**Affiliations:** ^1^ JC School of Public Health and Primary Care Faculty of Medicine The Chinese University of Hong Kong Hong Kong SAR China; ^2^ Centre for Health Systems and Policy Research The Chinese University of Hong Kong Hong Kong SAR China; ^3^ Shenzhen Research Institute The Chinese University of Hong Kong Shenzhen China; ^4^ Leeds Institute of Health Sciences University of Leeds Leeds UK; ^5^ Saw Swee Hock School of Public Health National University of Singapore and National University Health System Singapore; ^6^ Fatima Jinnah Medical University Lahore Pakistan; ^7^ Genetech Laboratory Lahore Pakistan; ^8^ Yorkshire Regional Genetics Service Leeds NHS Teaching Hospitals Trust Leeds UK; ^9^ Anthropological and Ethnological Research Institute Fudan‐Harvard Medical Anthropology Collaborative Research Center School of Social Development and Public Policy Fudan University Shanghai China

**Keywords:** antenatal screening, China, cross‐cultural comparison, informed decision making, Q‐methodology, relational autonomy

## Abstract

**Background:**

Although an integral part of ethical and quality health care, little is known about the informed decision making of Chinese women with different socioeconomic backgrounds within the context of antenatal testing.

**Methods:**

To explore women's viewpoints on informed decision making regarding antenatal screening, a Q‐methodology study that combines both quantitative factor analysis and interviews was conducted between June 2016 and February 2017 in Shanghai and Duyun. A total of 169 women (84 Shanghai and 85 Duyun) participated in the study of 41 ranked statements along a Q‐sorting grid.

**Results:**

Using by‐person factor analysis, five distinct viewpoints are identified: (a) choice is shared with the partner/husband, but the mother has the right to make the final decision; (b) having antenatal tests is not about choice but about a mother's responsibility; (c) choice is a shared decision led primarily by the partner/husband and secondarily by the doctors; (d) choice should be made using the advice of doctors, but the decision should be made with the partner/husband; and (e) choice is a responsibility shared with the partner, family and doctors.

**Conclusions:**

The study reveals that women with better education and higher incomes demonstrate more autonomy than those with less education. The nuclear family clearly emerges as the main decision makers in health‐care services in China.

**Patient and Public Contribution:**

The 169 participants shared their views and stories for at least an hour. They were debriefed after the interviews and contributed their thoughts on our study design and interpretation of the data.

## 1 INTRODUCTION

Antenatal screening tests refer to the use of a variety of medical and genomic technologies to help monitor the health conditions of the expectant mother and to check the health status of the foetus during pregnancy. Generally speaking, these tests include (a) an obstetric ultrasound; (b) blood tests; (c) non‐invasive prenatal screening technologies, such as maternal serum screening, nuchal translucency ultrasound and cell‐free DNA testing; and (d) invasive prenatal diagnostic testing technologies, such as amniocentesis and chorionic villus sampling. Through visualizing technologies and statistical models, antenatal testing calculates and presents the probability of having a child with congenital anomalies, which is factored into their reasoning on whether to keep the foetus and is believed to help relieve their ‘anxiety, fears and inner tensions’.^1^

In China, the importance of having antenatal tests is mentioned in numerous official guidelines on promoting maternal health care. By 2010, more than 500 health facilities in 29 provinces, autonomous regions and municipalities provide antenatal screening and diagnosis services. Although such tests are not mandatary, similar to parents in many other countries, most Chinese couples choose to have them. Since the implementation of the two‐child policy by the Chinese government in October 2015, an estimated 5.4 million additional births attributable to the new policy occurred.^2^ The demands for antenatal screening tests have increased correspondingly.^3^

According to the Maternal and Infant Health Care Law and other state‐issued guidelines for maternity care, obstetricians and gynaecologists must facilitate informed choice for antenatal screening to support patients to make a better decision.^4^ In one official announcement, the Chinese government specifically asks medical personnel to inform pregnant women with comprehensive and accurate information in accordance with the principles of medical ethics and to respect the pregnant woman's right to know and right to choose. Yet, very little is known about the views, perspectives and opinions regarding antenatal testing held by pregnant women in China, as well as what they are concerned with when making decisions in the face of positive results on foetal anomalies.

As a critical integral part of ethical health care, informed choice for antenatal screening has been internationally recognized and discussed.^5^, ^6^, ^7^, ^8^, ^9^, ^10^, ^11^, ^12^ Informed choice is based on relevant, high‐quality information about the advantages and disadvantages of all possible actions, allowing patients to make autonomous decisions.^13^, ^14^ However, this definition has been challenged in recent years. Some argue that the current definition is based only on an interpretation through the lens of biomedical ethics, and it neglects an understanding of the reality that multiple factors will contribute to the different understandings women hold concerning their choices.^15^, ^16^, ^17^, ^18^

Informed choice is embedded in the principle of autonomy where the chooser needs to act intentionally, with full understanding of the information and without other influences that affect their understanding.^19^ Some researchers argue that individual autonomy is incompatible with authority and cultural value.^20^ Beauchamp and Childress,^19^ however, point out that there is no full or complete autonomy but substantial autonomy in a practical world. Autonomy is in fact a matter of degree; in order for an action to be substantially autonomous, it must involve intentionality, understanding and voluntariness to an adequate extent. Furthermore, individual autonomy is defined differently across cultural contexts, and women in South Asia, for example, have little autonomy in the decision making of health‐care services compared with women in Western countries.^21^, ^22^

To make sense of the different understandings of informed choice from a woman's perspective, a series of cross‐culture studies have been conducted in recent years.^5^, ^23^ Women's understandings of informed choice can be divided into three categories: no autonomy, relational autonomy and individual autonomy. For women with no autonomy, the antenatal test is obligatory due to the responsibility for their foetus, the premise of being a good mother or religious reasons.^18^ Women with individual autonomy often make independent decisions, and such an interpretation distinctly reflects the emphasis of individual rights in the West.^5^

But, given the increasing emphasis on a shared‐decision model, informed choice is increasingly associated with relational autonomy.^24^ Relational autonomy means that individuals are embedded in and shaped within the context of social relationships and society.^25^ People may not recognize them as independent decision makers.^26^ For example, women from South Asia may prefer to follow the doctor's or husband's advice^5^, ^27^ and not make decisions themselves.^21^, ^22^ Previous research has also shown that Chinese women prefer to make an informed choice with their husband and family and want doctors to provide directive advice.^23^, ^28^ Within the Chinese society, an individualistic approach to autonomy would then fail to acknowledge how informed choice is never fully autonomous or overwhelmingly relational.

The traditional Chinese family is viewed as patriarchal, patrimonial, patrilineal and patrilocal, while women are subjected to subordination in social status, with the deeply rooted Confucius ideologies of Eastern societies contributing to the patriarchal nature of family systems.^29^, ^30^, ^31^ However, such a view tends to regard China as an entirely homogeneous society, which is far from accurate. China is a large country with an uneven distribution of economic development and a variety of cultures and values. Even though the modernization of China has brought a gradual homogenized lifestyle to the Chinese people, some minority and rural populations are maintaining their traditional culture and beliefs, which may also influence their interpretation of informed choice. As well, although the country as a whole has experienced substantial economic development and urbanization, a significant rural–urban disparity still exists. Thus, informed choice concerning antenatal screening tests, in the context of China, is bound by a set of social, economic and cultural norms.

Previous cross‐cultural studies have focused on whether the interpretation of informed choice of Western ideologies could be applied to other cultural societies, and how women from different cultures understand informed choice^5^, ^27^; past research has suggested different socioeconomic statuses may affect women's attitudes and participation in antenatal screenings as well.^32^ China presents a multiethnic population with a significant urban–rural gap, yet little is known about the interpretation of informed choice by Chinese women with different cultural, economic and ethnic backgrounds within the context of Chinese society. This study therefore aims to explore women's understandings of informed choice for antenatal screening tests in two different contexts in China: the first being in China's largest and most Westernized city, Shanghai, and the second being in a remote area of the western province of Guizhou, Duyun, which consists mostly of ethnic minorities (mostly Bouyei and Miao ethnicities).

## 2 METHODS

### 2.1 Participants

A purposive sampling method was used to recruit subjects. The inclusion criteria were Chinese women living in Shanghai and Duyun who had at least one child aged three years or younger. Recruitment flyers were posted and handed out by local research assistants in public places outside of hospitals, community maternal and child health centres, and other community centres in the two cities. In addition, invitations were sent out via the research team's personal networks. A total of 169 women (84 Shanghai and 85 Duyun) who fit the inclusion criteria and represented diverse backgrounds consented to participate and completed the study (see Table 1 for the demographic characteristics of the participants).

**Table 1:** Demographic characteristics of study participants (n = 169)

	Shanghai n = 84	Duyun n = 85
Participants’ education—N (%)***		
Below college	3 (3.6%)	49 (57.6%)
Equal or above college	81 (96.4%)	36 (42.4%)
Age in years—mean (SD)**	31.1 (3.9)	29.2 (4.8)
Participants’ employment status—N (%)***		
Full‐time employment	67 (79.8%)	36 (42.4%)
Housewife	15 (17.9%)	25 (29.4%)
Other	2 (2.4%)	24 (28.2%)
Household monthly income—mean (SD)***	31 832.5 (22 125.4)	7030.7 (5526.5)

** *P* < .01,

*** *P* < .001

### 2.2 Materials

**Figure 1:**
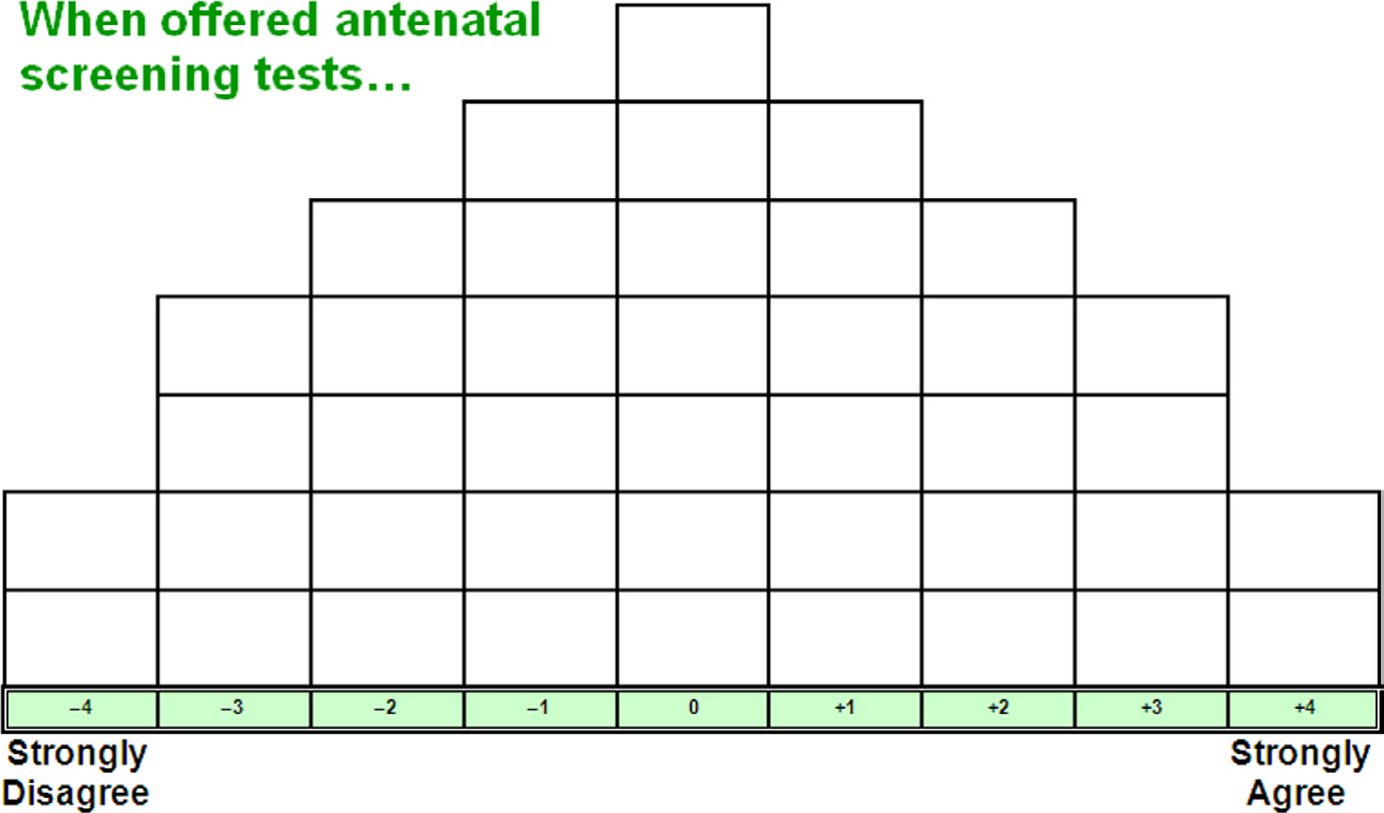
Q‐sorting grid and Q‐statements in Chinese

Q‐methodology was adopted to gauge the women's understanding of informed choice. Q‐methodology enables the study of subjectivity.^33^ Participants were asked to rank a set of 41 statements on informed choice along a Q‐sorting grid (see Figure 1 for the grid and the 41 statements), from the statements they agreed with the most to the least. The 41 statements (Q‐set) for this study had been previously developed for a UK research study,^5^ which included a Chinese version. To adapt the Q‐set to be more appropriate for the Chinese context, the research team compared the Chinese and English version used by the UK study, made minor revisions and pilot‐tested the revised Q‐set with five Chinese mothers. This Q‐set pushes participants to engage and consider the process of informed choice regarding antenatal screening. Each participant's distribution of the statements is called a Q‐sort.

### 2.3 Procedures

The study was conducted between June 2016 and February 2017 in Shanghai and Duyun. Ethical approval was obtained prior to the start of the study from a university ethics committee. Written consent was obtained from all participants before beginning the study. Q‐sorts were administrated individually in a location of the participant's choice (health centres, coffee shops or their homes). Participants were asked to read the statements and rank them from +4 (strongly agree) to −4 (strongly disagree). Then, they were asked to place each statement on the Q‐sorting grid. Each participant's sorting result was photographed. Then, a post‐sorting interview (audio‐taped) was conducted immediately to better understand the reasoning behind each participant's Q‐sort distribution. All post‐sorting interviews were transcribed in verbatim.

### 2.4 Analysis

Analysis of the Q‐sorts was conducted using PQmethod version 2.11. Factor analysis was used to correlate participants’ Q‐sorts to identify which participants’ Q‐sorts clustered together. Factors were extracted using principal component analysis, which maximizes similarities within factors and differences between them. Varimax rotation was used, which rotates factors to ensure that no Q‐sort loads significantly at the same level on more than one factor.^34^ Q‐sorts that were exemplars of each factor were identified, that is only Q‐sorts with a loading of ±0.04 (*P* < .01) on one factor.^34^ These exemplar's Q‐sorts were merged to create factor arrays (an average score for each item by factor).^35^ These factor arrays represent idealized Q‐sorts and are interpreted as different viewpoints (see Table 2 for the factor arrays).

**Table 2:** Factor arrays: scores against each statement by viewpoint

No.	Statement	V1	V2	V3	V4	V5
1	It's best to take one step at a time**—**to have the tests and not worry about what could happen	2	3	2	4	3
2	It is important for me to think about the challenge of bringing up a child with the condition	3	2	0	1	3
3	I think the offer of tests suggests people with these conditions are worth less than others	−1	0	−2	−4	0
4	I would worry about the child with the condition being treated badly by society	2	2	3	−4	3
5	I would look for what my religion says about having such testing	0	−3	−4	−3	−1
6	I would not discuss testing with anyone because the decision is mine alone	−2	0	−3	−2	−3
7	I would be angry if I was tested without being asked for my permission	3	−1	2	3	1
8	Doctors/midwives should give me their professional advice about whether to have testing	3	3	2	4	4
9	I would leave the decision about testing to doctors/midwives	−2	1	−3	1	2
10	If lots of other people are having testing, then testing would be fine by me	0	1	1	1	0
11	The decision about these tests is no more difficult to make than routine health tests in pregnancy, such as the mother's blood pressure or diabetes	1	1	−1	1	0
12	There is no decision for me to make because the tests are just part of good care for pregnant women	−1	4	1	2	−2
13	It is difficult for me to say ‘no’ to testing when doctors/midwives offer it	0	0	−4	0	1
14	I would take lots of time to make a decision about testing	0	−2	−2	−2	−1
15	Having too much information about the tests makes it difficult to make decisions	0	−1	0	−1	−1
16	I find it hard to make a decision about testing because there are too many decisions to make in pregnancy	−1	−3	1	0	−2
17	I would discuss it with my partner/husband but the decision would be mine.	3	1	0	1	0
18	I would not want to go against my partner/husband's wishes, so if we disagree, I would do what he wants	−1	−1	3	−2	0
19	Me and my partner/husband should make the decision about testing together	4	3	3	3	4
20	I would keep my in‐laws out of the process of making the decision about testing	1	1	−2	0	0
21	I would take advice from my parents or brothers/sisters about having the tests	0	−1	−1	0	1
22	My parents’ or brothers’/sisters’ views would sway my decision about testing	−1	−2	−3	−1	2
23	My in‐laws’ views would influence my decision about testing	−2	−3	−2	−3	−1
24	I think doctors/midwifes should give information only, not advice about whether to have testing	1	0	−1	−1	−3
25	I believe doctors/midwives would not offer the tests if it wasn't important to have them	0	2	1	2	3
26	I believe having these tests is just part of being a good mother	2	4	4	2	2
27	I would want information provided by doctors/midwives to help me make my decision about testing	2	2	1	3	2
28	I would consider myself fortunate to be offered these tests free of charge	1	3	0	0	−1
29	I would worry about what others might think if I decided to terminate a child	−3	−2	−3	−2	−1
30	I should not be asking the doctor or midwife to make a decision about whether or not I have testing	1	−1	1	2	−2
31	I value the opportunity to think about termination of a child with a condition	2	2	3	2	0
32	If I cannot decide whether to have testing then I should not be tested	1	0	0	−1	−3
33	Decisions about testing should only be made after carefully thinking through all the possible consequences of testing	4	0	2	3	1
34	I would worry about people judging me as being irresponsible if I decide not to have testing.	−3	−1	−1	0	1
35	I would not have an abortion, so there's no point in having testing	−4	−3	−1	−3	−4
36	I would accept the child that God gives me so there is no reason to have testing	−4	−4	−2	−3	−4
37	I want information about the tests but I do not want to make the decision	−2	−2	0	−1	−2
38	I do not want information from doctors/midwifes—I will use my own judgment	−2	−4	−1	−2	−3
39	My partner/husband should make the decision about testing	−1	0	4	−1	2
40	Doctors should tell me what to do, not ask me to make the decision about testing	−3	1	2	1	1
41	I prefer not to make the decision about testing because I am scared of making the wrong decision	−3	−2	0	0	−2

Eight factors were originally extracted, with an eigenvalue of 1.00 or more^35^ and at least one exemplar.^36^ A five‐factor solution was reached after inspection of factors 6 to 7 showed that they did not provide distinct viewpoints that were not captured in the other factors. These five factors explain 62% of the total variance.

Interpretations of the five factors were carried out by examining and comparing the factor arrays, with a particular focus on statements in the ‘strongly agree’ and ‘strongly disagree’ columns. Statements identified as statistically distinguishing each factor were used to interpret factors by comparing the position of the ‘between’ factors. In addition, consensus statements on which the levels of agreement/disagreement were similar across factors were also analysed. Transcriptions from the post‐sorting interviews were read repeatedly and used to inform, confirm or further clarify the participants’ sorting results. Since these interviews were quite short, no specific coding schemes were used. Initial interpretation was conducted by the first author, and then subsequently discussed with the other authors and continually examined against the qualitative data collected.

## 3 FINDINGS

The study participants included 84 women from Shanghai and 85 from Duyun. Most of the participants from Shanghai had higher education levels and family income than those from Duyun. The average age for the five factors was 30 years, with a slight difference between the oldest cohort (factor 1, average 31 years) and the youngest (factor 3, average 27 years) (see Table 3 for details on participants’ demographic information for exemplars in the five viewpoints).

**Table 3:** Demographic information for exemplars in the five factors/viewpoints

	Viewpoint 1 exemplars n = 21		Viewpoint 2 exemplars n = 22		Viewpoint 3 exemplars n = 6		Viewpoint 4 exemplars n = 11		Viewpoint 5 exemplars n = 9	
	Shanghai	Duyun	Shanghai	Duyun	Shanghai	Duyun	Shanghai	Duyun	Shanghai	Duyun
No. of exemplars	16	5	5	17	2	4	6	5	7	2
Age in years—mean (SD)	31.8 (3.1)	31.0 (7.4)	33.8 (2.8)	29.5 (4.0)	31.5 (7.8)	25.3 (1.7)	29.7 (2.6)	28.2 (2.6)	30.3 (4.0)	23.5 (0.7)
Household monthly income (RMB)—mean (SD)	244 499.2 (9135.8)	14 339.6 (19 960.9)	24 399.0 (13 464.8)	7822.8 (1845.2)	24 999.0 (22 627.4)	6750.0 (3774.9)	25 832.7 (12 122.8)	5999.4 (2915.7)	32 570.7 (31 079.6)	4499.0 (707.1)
Participants’ employment status—N (%)										
Full‐time employment	15 (93.8)	2 (40%)	3 (60%)	8 (47.1%)	2 (100%)	0	6 (100%)	0	7 (100%)	0
Housewife	1 (6.3%)	1 (20%)	2 (40%)	5 (29.4%)	0	2 (50%)	0	2 (40%)	0	1 (50%)
Other	0	2 (20%)	0	4 (23.6%)	0	2 (50%)	0	3 (60%)	0	1 (50%)
Participants’ education—N (%)										
Below college	0	1 (20%)	1 (20%)	9 (52.9%)	1 (50%)	3 (75%)	0	4 (80%)	0	1 (50%)
Equal or above college	16 (100%)	4 (80%)	4 (80%)	8 (47.1%)	1 (50%)	1 (25%)	6 (100%)	1 (20%)	7 (100%)	1 (50%)

One consensually agreed statement emerged across all of the viewpoints: (#19) ‘Me and my partner/husband should make the decision about testing together’.

Participants emphasized the importance of shared decision making with their husband/partner (+4 in factors 1 and 5; +3 in factors 2, 3, 4), indicating the mutual responsibility of parenthood and of a healthy child:

*The child belongs to us, not only me alone. So, my husband and I should take all the responsibility from the time that I am pregnant to the birth of the child*. (DY090)

The findings should be read in the light of this insight.

**Figure 2:**
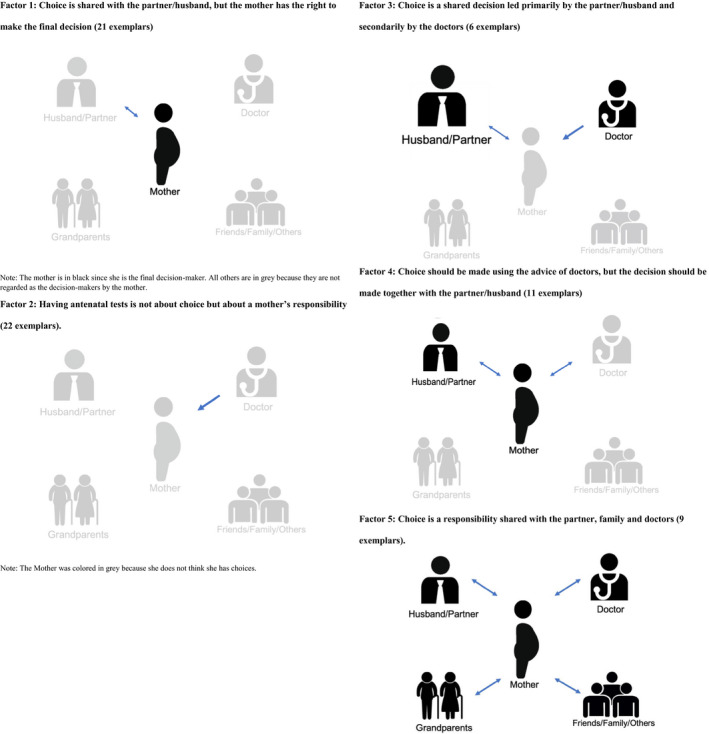
Visual illustrations of the five factors. Factor 1: Choice is shared with the partner/husband, but the mother has the right to make the final decision (21 exemplars). Note: The mother is in black since she is the final decision maker. All others are in grey because they are not regarded as the decision makers by the mother. Factor 2: Having antenatal tests is not about choice but about a mother's responsibility (22 exemplars). Note: The mother was coloured in grey because she does not think she has choices. Factor 3: Choice is a shared decision led primarily by the partner/husband and secondarily by the doctors (6 exemplars). Factor 4: Choice should be made using the advice of doctors, but the decision should be made together with the partner/husband (11 exemplars). Factor 5: Choice is a responsibility shared with the partner, family and doctors (9 exemplars).

Despite the similarities, some unique features emerged across the viewpoints: choice as shared with the partner/husband, but with the mother's final decision; choice as a mother's responsibility; choice as shared decision yet led by the partner/husband and secondarily by the doctors; choice as advised by doctors, but made together with the partner/husband; and choice as a responsibility shared with the partner, family and doctors. Visual illustrations of the five factors are provided in the Appendix S1 (Figure 2).

### 3.1 Factor 1: Choice is shared with the partner/husband, but the mother has the right to make the final decision (21 exemplars)

This viewpoint was held by 21 participants: 16 from Shanghai and 5 from Duyun. Most of these participants had higher educational attainment and income levels compared with participants from the other factors.

In this viewpoint, participants emphasize the mother's autonomy; they believe the decision should be led by the mother (#17, +3), and conversely, they disagree with the husband/partner taking the lead in the process (#39, −1). Nevertheless, in line with a relational approach to autonomy, participants strongly agree that decision making about testing should be shared with the partner/husband (#19, +4), stressing the mutual responsibility of parenthood. Informed choice is then shared with the partner, yet led by the mother.

*It was necessary to discuss with him, but since I was the pregnant one, the final decision is mine*. (SH004)

*The pregnancy was my own business. … Sometimes the family discussion makes things worse … Sometimes the doctor asked me whether I want a boy or girl, I said no matter what the gender of baby was, and he/she was my child. I personally feel all those things depend on my decision*. (DY030)

In line with autonomous decision making, participants believe that doctors and midwives should provide information rather than advice (#24, +1). They strongly disagree with a directive approach by doctors and do not want to be told what to do (#40, −3). Accordingly, they would be angry if tested without permission (#7, +3). Moreover, participants do not seem to agree that doctors would always act in patients’ best interest (#25, 0). Accounts suggest both a desire for autonomy and a feeling of mistrust towards doctors may lead to their exclusion from decision making.

*An individual is the master of their own body. The doctor is only allowed to give you some piece of advice, but not make any decision*. (SH003)

*We are not professional medical staff so that sometimes we are unable to know the relevant knowledge, and we surely worried about the health of babies. I will carefully consider the examinations before accepting them* (DY017).

*I do not trust doctors according to my previous experiences, so I’ll never allow the doctor to make a decision for me. I am the mother who can make this decision. I would like to consider the advice from the doctor, but I definitely will not ask him to the decision* (DY017).

Also consistent with relational autonomy, participants strongly disagree with ‘I prefer not to make the decision about testing because I am scared of making the wrong decision’ (#41, −3) and ‘I would worry about people judging me as being irresponsible if I decide not to have testing’ (#34, −3); they agree instead with taking enough time to think carefully about the effects of testing (#33, +4).

I *must consider it clearly, after all, it is an important thing for me. I do not make the decision lightly*. (SH057)

Participants’ Q‐sorts also show the importance of antenatal testing to them (#35, −4; #36, −4). In particular, participants would consider termination of a child with a condition (meaning any type of foetal abnormalities) (#31, +2), the challenges that raising such a child may cause (#2, +3) and the discrimination the baby may face (#4, +2).

*If the child is indeed unhealthy, or is probably a burden on both your family and life in the future, I think I might choose abortion*. (DY045)

*Sure, in China, children with conditions are discriminated, which is a result of the social environment*. (SH022)

### 3.2 Factor 2: Having antenatal tests is not about choice but about a mother's responsibility (22 exemplars)

This factor had 22 exemplars: 17 from Duyun and 5 from Shanghai. Most of the participants from Duyun had a lower education level and lower family income than those from Shanghai. Overall, participants in this factor had the lowest level of income and the second lowest level of education after factor 3.

In this viewpoint, participants emphasize testing as a maternal responsibility rather than a choice.

Participants strongly believe that having the antenatal tests is ‘part of good care for pregnant women’ (#12, +4) and is ‘part of being a good mother’ (#26, +4). Testing appears to be a maternal duty rather than a decision, since it fulfils the mother's responsibility for ensuring both the mother's health and the child's health. Accordingly, the decision about testing is not considered hard (#16, −3) and does not necessarily involve careful thinking about the effects of testing (#33, 0).

*Doing the antenatal examinations means that you are responsible for yourself and the child. So, you were a good mother. If you give birth to an unhealthy baby, how could you be a good mother?* (DY031)

In contrast with viewpoint 1, participants would not be angry if they were tested without permission (#7, −1). Accounts showed in fact that they value a more directive approach by doctors, and trusted doctors’ advice (#25, +2), and would conversely play a more passive role (#38, −4).

I *trust doctors. I would not have any doubts because I think doctors’ advice is best for me, otherwise, the doctor won't ask me to do the tests*. (DY025)

*Following the suggestion of the doctor to take care baby is much better, at least, it will more accurate than my own judgment*. (SH012)

Participants strongly disagree with ‘I would accept the child that God gives me’ (#36, −4), confirming the importance of antenatal testing. This viewpoint is also the one where the possibility of having tests free of charge is most welcomed (#28, +3). Similar to the previous viewpoint, participants would consider termination of a child with a condition (#35, −3; #31, +2), think about the discrimination he/she may face (#4, +2) and the burden of raising such a child (#2, +2) both for the family and for the society.

*First thing to do is taking antenatal checking, if the child does not have any problem I will certainly keep him/her. But if the child really has a problem, he really will place a great burden on the whole family. It is also miserable for children. So I think we must think about it carefully*. (DY069)

*I should take responsibility for me and for society. If I am not doing the examination, there is a possibility that the baby born with defect, which is a huge burden both for my family and society. Thus, it is very important*. (DY018)

### 3.3 Factor 3: Choice is a shared decision led primarily by the partner/husband and secondarily by the doctors (6 exemplars)

This factor had 6 women: 4 from Duyun and 2 from Shanghai. The participants from Duyun had a lower family income than those from Shanghai, but their educational levels were the lowest compared with other factors.

The emphasis of this viewpoint is on the leading role of the partner/husband in decision making and on a directive approach by doctors. Participants strongly agree with the partner making the decision about testing (#39, +4) and with delegating to him the choice in case of disagreement (#18, +3). Although participants acknowledge the decision should be shared with the partner/husband (#19, +3), they believe he should have the final say, thus displaying lower perception of their autonomy in decision making.

As in viewpoint 2, participants value professional medical advice (#8, +2), would accept information to guide their decision (#27, +1) and welcome a more directive approach, where doctors should tell the mother what to do (#40, +2). Participants believe the decision about these tests is more difficult than other routine tests during pregnancy (#11, −1); additionally, they expressed a neutral position to ‘I want information about the tests but I do not want to make the decision’ (#37, 0). This uncertainty and struggle with the decision may possibly explain why they welcome medical guidance. Also, similar to factor 2, this viewpoint has the lowest income and education level, which may be related to the participants’ attitudes towards directive medical guidance.

*When you come to see the doctors, they will give you good advice. I will do whatever the doctor tells me to do, because the doctor gives the best advice*. (DY048)

*Taking prenatal examination is decided by doctors. It is nonsense to take others’ advices, because they cannot guarantee anything*. (SH064)

Yet, although participants value doctors’ professional advice, they strongly disagree with ‘it is difficult for me to say “no” to testing when doctors/midwives offer it’ (#13, −4). Participants’ accounts show some ambiguity, where they acknowledge the doctors’ authority and yet feel constrained by it; they would challenge the doctors’ opinion if they believe tests are unnecessary.

*The advice of the doctor is so authoritative that I find it is hard to say no. He is professional and he can tell me some relevant knowledge. But if there is indeed no need to do some examinations … I will refuse to do it*. (DY098)

*The doctor always asked me to do a lot of tests. Sometimes, I didn't want to do it, but he said I must to do. I felt kind of being forced. If I refused to do it, the doctor would say that if I didn't do the test, there was nothing I could do if the baby was unhealthy. It sounded kind of threatening*. (DY029)

In comparison with previous viewpoints, family—in particular in‐laws—would not be completely excluded from the decision process (#20, −2), yet family members would not influence the final choice (#22, −3; #23, −2).

I *think this is my own business, so my opinion accounts for dominant. I rarely listen to my parents’ opinions*. (DY098)

Participants place no emphasis at all on religion (#5, −4) and disagree with ‘I would accept the child that God gives me’ (#36, −2), thus stressing religious considerations would not affect their choices. They emphasize instead the importance of antenatal testing for an informed decision; similar to other factors, they take into consideration the possibility of abortion (#35, −1) and the discrimination a child with a condition may face (#4, +3). They nevertheless disagree with the idea that ‘the offer of tests suggests that people with these conditions are worth less than others’ (#3, −2).

*… I think we must do testing. When you are pregnant, you must do the test*. (DY048)

*If your child has a condition… he will be mocked and discriminated against*. (DY029)

### 3.4 Factor 4: Choice should be made using the advice of doctors, but the decision should be made together with the partner/husband (11 exemplars)

This factor had 11 exemplars: 6 from Shanghai and 5 from Duyun. Participants from Shanghai had higher educational levels and income than those from Duyun.

The emphasis in this viewpoint is on decision making as a process advised by doctors and shared with the partner/husband.

In contrast with viewpoint 3, participants believe the husband should not take the lead in the process of decision making (#39, −1), and it should instead be shared and led by the two parents together (#19, +3).

*The most important family members are the baby’s parents, so the decision should be made by them together*. (SH025)

Similar to factors 2 and 3, participants highly value both medical information (#27, +3) and advice (#8, +4) to guide their decisions about testing. However, in contrast to viewpoint 2, they would be angry if the test was done without their permission (#7, +3), displaying a less passive role.

*The doctor is professional medical staff, and I believe that their suggestions are very important. I trust doctors*. (SH008)

*In addition to my personal subjective views, I think the doctor’s advice is very important and professional. Although we can find a variety of information on the Internet, the doctor's advice is the most direct and authoritative*. (SH025)

*…you are a doctor, you have a professional knowledge. We come to find the doctors and ask for help because we know nothing. If we have such medical knowledge, we will no longer need doctors. I totally disagree with the doctor asking me to do some checks without my permission* (DY037).

Although they agree about the importance of testing (#35, −3; #36, −3), compared with other viewpoints, participants strongly disagree with ‘worry about the child with the condition being treated badly by society’ (#4, −4). Similar to factor 3 only, they disagree with ‘the offer of tests suggests that people with these conditions are worth less than others’ (#3, −4). Accounts in this viewpoint display the need for acceptance of any children, regardless of their conditions.

*I think each person has their own characters and thoughts. Why do you despise other children or compare your children to others? Although at this time your children are not as good as others, it may change in the future. So, I disagree with it*. (DY037)

*Every child is equal, each one is your own. There is nothing to worry about*. (DY039)

*He is my child. For me, he is the best even if he is discriminated by whole the society*. (DY043)

### 3.5 Factor 5: Choice is a responsibility shared with the partner, family and doctors (9 exemplars)

This factor had 9 exemplars: 7 from Shanghai and 2 from Duyun. Participants had the highest average income and educational level, yet participants from Shanghai had higher levels than those from Duyun.

A distinctive feature of this viewpoint is the shared nature of decision making, where the partner, family members and doctors are all perceived to have different roles. Rather than lacking autonomy, this viewpoint seems to be aware of the responsibility involved, and the participants are thus willing to share that responsibility.

Firstly, this is the only account where parents and siblings would be involved in the decision making and could influence it (#22, +2); in‐laws instead would not be so actively involved (#23, −1).

*… The baby belongs to the whole family. My husband and my parents also need to be responsible for him/her*. (SH005)

*I think the whole family should discuss about what kind of test need to do. And everyone should have a complete understanding about it, which is very important. If there is any problem, we can face it together*. (SH023)

Secondly, participants value professional medical advice (#8, +4) rather than information alone (#24, −3). Similar to viewpoint 2, they welcome a more directive approach by doctors/midwives, to whom they agree they would leave the decision about testing (#9, +2).

I *think the doctor has the right to give advice. I will listen to the doctor* (SH062).

Thirdly, similar to viewpoint 3 only, participants agree ‘the partner/husband should make the decision about testing’ (#39, +2), thus adopting a more passive role for themselves.

Participants agree that decisions about testing required careful thinking about the effects (#33, +1) and would involve several subjects to guide their choice. They are particularly concerned about being blamed as irresponsible if they were not tested (#34, +1).

Similar to viewpoint 1, they value testing highly (#32, −3; #36, −4; #36, −4) and would consider abortion (#31, 0) without being concerned about people's judgement (#29, −1). In particular, participants worry about children with conditions being discriminated (#4, +3) against and the challenges of raising them (#2, +3).

*I think testing is necessary. If you don’t do it … you are not being responsible for the child* (DY087)

*I worry about the discrimination against my child… He will be mocked by others so I worry about this…I think all the parents have the same idea that it is a serious burden for a family to raise such a child*. (DY099)

*The only way to know about the baby’s health is through a series of antenatal tests. Giving birth to a child with conditions will bring him/her a lot of pain*. (SH005)

## 4 DISCUSSION

The purpose of this study is to explore women's understandings of informed choice concerning antenatal screening tests in two different societal contexts in China. The findings show that women interpret informed choice in different ways, supporting the results of past studies.^5^, ^23^, ^27^ We found five viewpoints in our study. Participants’ preferences for making informed choices about antenatal screening ranged from decision making by the doctor, their husband or their larger family unit, including themselves as the final decision maker. These differing views show that even within a collectivist society, informed choice as an individual choice is important to women, as it is in Western society. In contrast to other Western societies, participants in all factors show a willingness to terminate the pregnancy when the probability of the foetus having a physical or mental disability was high. This may be partly due to the absence of participants claiming any religious belief, where many religions are believed to forbid abortion, coupled with less taboo or restrictions regarding the termination of pregnancy under the current official guidelines. This contrasts with what emerged in similar studies conducted in other societies,^4^ where choice was informed by religious beliefs and values, and therefore, abortion would not be an available option.

Factors were not city‐specific, since women from both Shanghai and Duyun were present in all five viewpoints. However, women in factors 1 and 5 were mainly from Shanghai (76% and 78%, respectively). Factors 2 and 3 were more representative of Duyun (77% and 67%, respectively). Factor 4 was more balanced, yet slightly more representative of women from Shanghai. Women in Shanghai had generally higher education and income levels than those in Duyun.

A dominant theme that prevailed across all five factors was that all women would involve the partner–husband in decision making, thus confirming the relevance of a relational approach to autonomy in the Chinese context.^23^ Such a view, as emerged from all 169 participants of this study, is shaped by a social context where the individual is interdependent to her family. The husband/partner, family members and health‐care workers as well co‐participate in the decision‐making process to different degrees. This differs from what has been found in similar studies conducted in Western contexts,^4^ where choice emerged instead as a mother's individual right and women showed little interest in involving other people in the decision process. In the Chinese context, autonomy is a matter of degree.

Our data suggest a possible relation between the degree of autonomy and level of education and income, exemplified by slightly different accounts by women in Shanghai and Duyun. Participants in factor 1 display the highest autonomy; for them, choice would be shared with the partner/husband, yet the woman had the right to make the final decision. This cohort was from Shanghai and had comparatively high education and income levels. Women in factors 2 and 3 were more representative of Duyun and ranked the lowest in terms of education and income level. Their attitudes towards informed choice are rather passive. Women in factor 4, also slightly more representative of Shanghai, believe choice should be made together with the husband. Factor 5 was the one ranking the highest in terms of education and income level and mainly representative of Shanghai. Women in this factor agree both family and the partner/husband could influence their choice. Yet, more than a withdrawal from choice, their viewpoint indicates the acknowledgement of other subjects’ points of view and an awareness of the responsibility involved in decision making.

Participants rarely mentioned the involvement of family at large in decision about testing. This is surprising especially in a society where family ties are deemed to be fundamental. Only women in factor 5 would involve parents, siblings and in‐laws and let them influence their choice; participants in factor 3 would not exclude in‐laws from the process, yet they would not sway the parental couple's decision. All the other factors would not include any family members at all. Instead, the focus would be on the couples. These findings reflect an undergoing transformation of contemporary familial relations in Chinese society that other scholars have discussed.^37^ Yet, the emphasis on the nuclear family as decision makers emerged from our findings cannot be simply understood as a shift towards individualism and a collapse of family bonds and filial piety; filial relations are in fact being re‐negotiated both by adult–children and by parents. Our research shows in this transformational process decisions about antenatal testing do not fall under the authority of the family at large, but are conceived instead as a parental couple's choice. Our findings are in line with the recommendations of previous studies conducted in China, Pakistan and Hong Kong^22^ and suggest that within the Chinese context health professionals should facilitate the partner/husband's participation in decision making about antenatal tests, while involving other family members with extreme caution in the process.

Similar to previous research,^5^, ^23^ all participants acknowledge the role of medical professionals in informed choice about prenatal testing. Yet, participants’ accounts range from acknowledging the need for information, through seeking advice, to embracing more directive approaches. Since this research focused on Chinese society specifically, different attitudes towards doctors must be understood and contextualized within the shifting role of health professionals in Chinese control population policies specifically.

Policy implications of this research need to be stated within the context of contemporary China. Starting from the 1980s, the improvement of the quality (*suzhi* 素质) of the population has been crucial in Chinese modernization project.^38^ In an effort to promote a quality population, medical professionals have acted as ‘quality inspectors’.^39^ They have tended to adopt a directive role, openly promoting antenatal tests and discouraging the birth of a ‘defective’ child, that would be an economic burden for the family.^34^ With the introduction of the two‐child policy in 2015, there has been a shift in discourse towards an emphasis on quantity of births. The promotion of antenatal genetic testing is also meant to promote the quality of population. However, the results of the antenatal tests are expressed in terms of risk rather than certainty of diagnosis. To avoid any blame in the case of a wrong diagnosis and shift of responsibility onto parents, health professionals have tended to adopt a less directive approach, aiming to provide information rather than advice, and leave informed choice up to the family.

Our study shows that some Chinese women seem to have embraced this recent discourse centred on informed choice. For doctors and midwives, it is important to be aware of the expectations of the mothers who regard medical professionals as merely information providers. Meanwhile, it is equally important to notice how the emerging non‐directive discourse in genetic counselling is likely to create frustration among women.^39^ Some Chinese women still prefer guidance from doctors. This attitude is also in line with what emerged from previous research conducted in non‐Western societies, where a more directive approach was welcomed.^22^ Our data suggest a possible relation between lower educational or income level and the acceptance of a more directive approach from medical professionals, exemplified by the different positions of women in Duyun and Shanghai. Hence, when providing advice and information on antenatal testing results, the medical professionals need to take the patients’ education, socio‐economic backgrounds into account and try to seek a balance between the non‐directive and directive approaches. Further research should investigate this tension and address how to better facilitate informed choice for antenatal genetic testing in the Chinese context.

Another interesting finding is that, in some form, all participants display a sense of responsibility to ensure a healthy birth by utilizing antenatal testing. The individual and the state discourse seem to overlap. Birth in China is a ‘state affair’.^38^ Entering the antenatal health‐care system is equated with women embodying the state discourse of a quality population, hence each individual's responsibility for a healthy birth (*yousheng*). Some of our participants seem to strongly embrace this discourse; for them, having antenatal tests is rather a mother's responsibility than a choice. Getting tested fulfils the maternal duty of ensuring both the mother's health and child's health. Additionally, women in all the factors acknowledge antenatal testing as an essential tool for informed choice; all would take into consideration termination of a child with a condition, unlike Western populations.^5^, ^40^, ^41^ Many of them worry about the discrimination a child with a condition would face, and all would be concerned about the burden such a child would cause to the family. This may relate to the widespread stigma towards disability in Chinese society, the lack of appropriate economic and social support for families, and the consequent challenges they would face.^40^ Our findings suggest that without placing enough resources towards disability rights, informed choice in antenatal testing is restricted by societal values of the ‘right choice’. Hence, it is important for policymakers to consider improving the rights of people with disabilities and enhancing public awareness of such rights, to provide more social support to families with children with disabilities and to greatly promote barrier‐free facilities, so that when the Chinese mothers are making decisions, their decisions are less affected by such concerns.

## 5 LIMITATIONS

The statements used in the study were not developed anew but translated from English to Chinese from previous research. When the Chinese version was used during the interview, women with lower education levels had difficulty understanding the statements and researchers had to provide further explanation. Also, the study was only conducted in Duyun and Shanghai, and the conclusions may not be representative of or generalizable to the Chinese population as a whole. In addition, due to the limited time and resources for this research, the participants might not have been diverse enough to represent women with all possible social economic and cultural backgrounds from these two cities. Meanwhile, for the Q‐method, our sample size might have been too large to differentiate between the viewpoints. Hence, it raises concerns on noise in the factors, which might have led to misinterpretation.

## 6 CONCLUSION

This study addressed a series of factors that exert influence on women's informed choice regarding antenatal tests in China. The findings show that most women regard informed choice as a shared decision with their husband. Information provided by the medical professional enables them to make a better decision. The understanding of shared decision making is not only explained with the conception of relational autonomy, but also a way to balance the relationship between women and their family members, society and the authority represented by the doctors and relative national fertility policies. Our study is the first in‐depth research about the subjective understanding of informed choice in Chinese women. The participant's educational level and income level are possible distinctive factors representing two different populations in China. This study suggests further consideration of the process of informed choice to improve the health‐care policies protecting child and maternal health.

## CONFLICT OF INTEREST

All the authors declare no conflict of interest.

## AUTHOR CONTRIBUTIONS

DD, SA, HY, HJ, YR, MA and JZ made substantial contribution to the conceptualization, design and research protocol for the study. DD and JZ made substantial contributions to data collection and interpretation. DD, EN and JZ wrote the first draft of the manuscript. All authors were involved in revising the article and approved the final manuscript.

## ETHICAL APPROVAL

The study was approved by the Committee on the Use of Human and Animal Subjects in Teaching and Research of Hong Kong Baptist University, which the first author worked for when the study was conducted. The ethics approval code is FRG1/15‐16/057.

## Supporting information

Appendix S1

## Data Availability

The data that support the findings of this study are available from the corresponding author upon reasonable request.
