# Supplementary material for: Decision making on antenatal screening results: A comparative Q‐method study of women from two Chinese cities
Source: Health Expect. 2020 Dec 14;24(2):363–76. doi: 10.1111/hex.13178 (PMC8077134; doi:10.1111/hex.13178)
Supplement: Supplementary file 1 — Appendix S1 [file HEX-24-363-s001.doc]

| 1. 最好是一步一步来 —— 先做检测而不是去担心检测之后可能会发生的事。 |
| --- |
| 1. 对我来说，重要的是要想清楚，抚养一个有问题的婴儿可能会带来怎样的挑战。 |
| 1. 我认为，提供这些检测说明，有这些问题的人跟正常人比起来，他的社会价值会低一些。 |
| 4. 我很担心有问题的孩子会遭到社会的歧视 |
| 5. 我会查看我所信仰的宗教对这些检测的说法。 |
| 6. 关于是否做这些检测，我不会和其他人商量的，因为这应该是我一个人的决定。 |
| 7. 如果在没有事先经过我的同意的情况下进行了检测，我会很生气。 |
| 8. 医生应该在是否接受某个检测的问题上向我提供他们的专业建议。 |
| 9. 我会把是否接受某个检测的决定留给医生来做。 |
| 10. 如果很多其他人都接受这个检测，那么我也接受。 |
| 11. 关于是否做这些检测的决定并不比做其它诸如血压、血糖之类常规孕期检测的决定要难。 |
| 12. 我不需要对这些检测进行考虑，因为它们都是对孕妇提供的良好呵护的一部分。 |
| 13. 当医生向我提供某个检测时，我觉得很难说‘不 ’。 |
| 14. 我会花很长的时间来决定是否做这些检测。 |
| 15. 太多关于这些检测的信息会增加做决定的难度。 |
| 16. 我觉得对这些检测作决定很难，因为在怀孕的过程中有太多的决定要做。 |
| 17. 我会和我丈夫/爱人商量，但是最终的决定权是我的。 |
| 18. 我不会反对我丈夫/爱人的意愿。如果我们意见相左，我会遵循他的意愿。 |
| 19. 我和我的丈夫/爱人应该一起做关于检测的决定。 |
| 20. 我不会让我的公婆参与到与这些检测有关的决定当中。 |
| 21. 我会征求我的父母/兄弟姐妹对于这些检测的建议。 |
| 22. 我父母/兄弟姐妹的看法会影响到我对这些检测的决定。 |
| 23. 我公婆的看法会影响到我对这些检测的决定。 |
| 24. 我认为医生应该只提供相关的信息，而不应该对是否接受检测提出建议。 |
| 25. 我相信如果这些检测不重要的话，医生是不会跟我提及的。 |
| 26. 我认为接受这些检测是做一个好母亲的一部分。 |
| 27. 我需要参考医生提供的信息来帮我做这个决定。 |
| 28. 我觉得如果有人向我免费提供这些检测是件很幸运的事。 |
| 29. 如果我决定终止怀孕，我会担心别人对此的看法。 |
| 30. 我不应该要求医生来做对这些检测的决定。 |
| 31. 我很珍惜有这样一个机会来思考，如果婴儿有问题，是否要终止怀孕利。 |
| 32. 如果我不能对这些检测做出决定，那么我就不该被检测。 |
| 33. 只有在慎重考虑过检测会带来的所有可能的后果之后，我们才应该对这些检测作决定。 |
| 34. 如果我决定不做这些检测，我担心其他人会认为我是不负责任的。 |
| 35. 无论怎样我都不会选择人工流产，所以我认为没有作这些检测的必要。 |
| 36. 无论怎样我都会接受这个上天赐给我的婴儿，所以我认为根本没有作检测的必要。 |
| 37. 我想了解关于这些检测的信息， 但是，我不想对这些检测作决定。 |
| 38. 我不需要医生提供信息 —— 我会根据自己的判断作出决定。 |
| 39. 我丈夫/爱人应该对这些检测作决定。 |
| 40. 医生应该告诉我做什么，而不是让我自己来做这些决定。 |
| 41. 我不愿意对这些检测作决定，因为我怕做出错误的决定。 |

Fig 1: Visual illustrations of the five factors

**Factor 1:** **Choice is shared with the partner/husband, but the mother has the right to make the final decision (21 exemplars)**


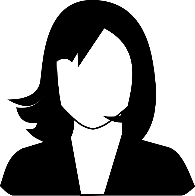

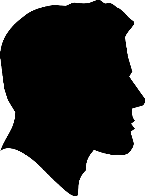

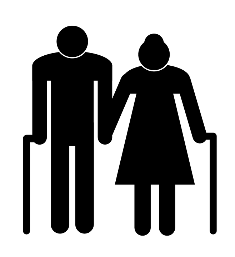

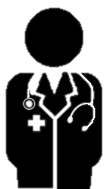

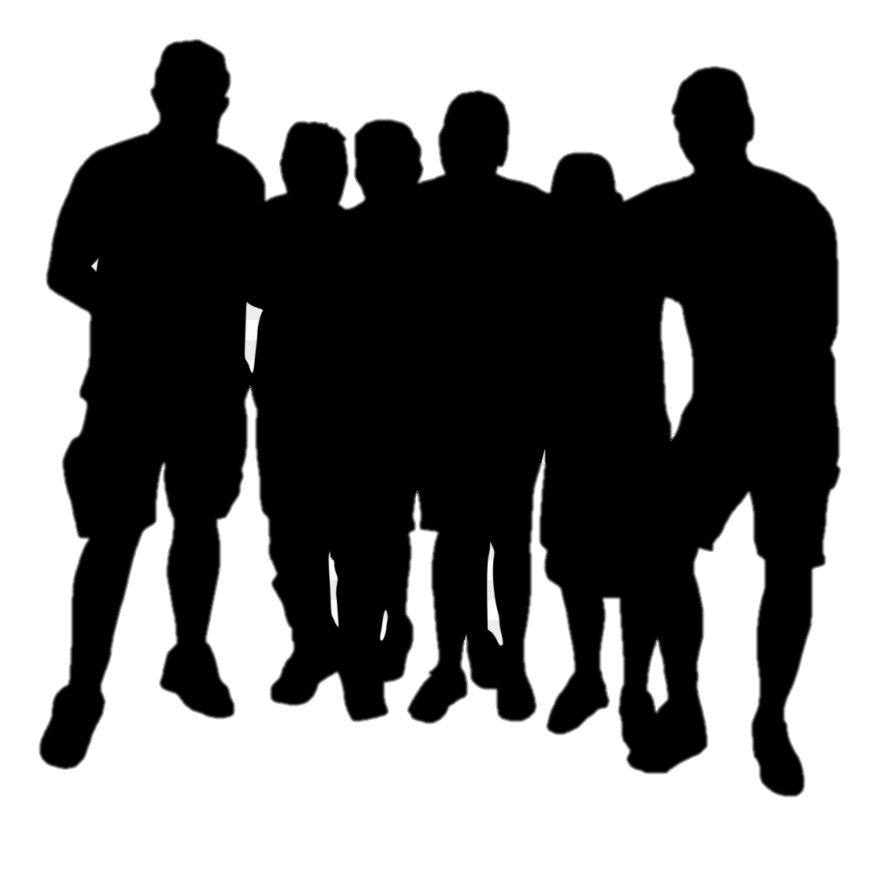


Mother

Husband

/Partner

Doctor

Grandparents

Friends/Families/Others

Note: The mother is in black since she is the final decision-maker. All others are in grey because they are not regarded as the decision-makers by the mother.

**Factor 2: Having antenatal tests is not about choice but about a mother’s responsibility (22 exemplars).**


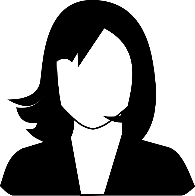

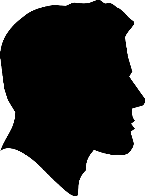

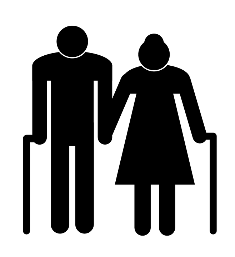

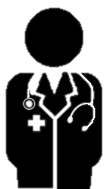

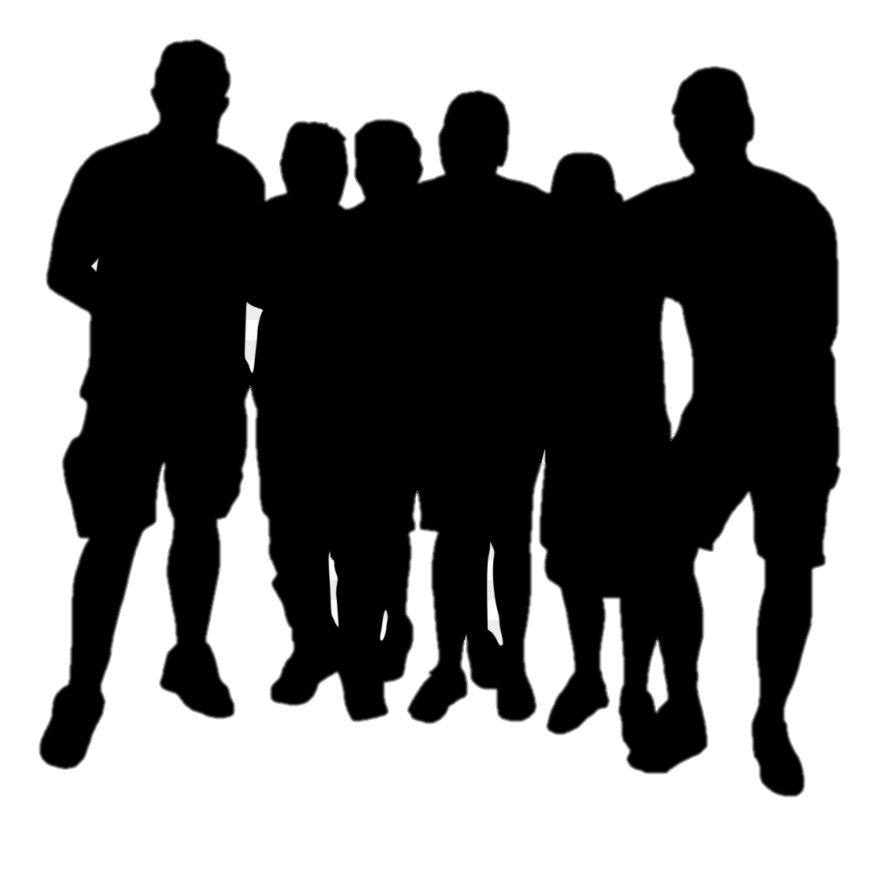


Mother

Grandparents

Friends/Families/Others

Doctor

Husband

/Partner

Note: The Mother was colored in grey because she does not think she has choices.

**Factor 3: Choice is a shared decision led primarily by the partner/husband and secondarily by the doctors (6 exemplars)**


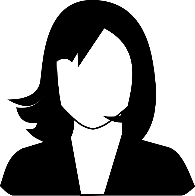

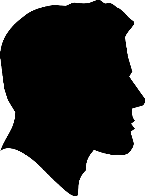

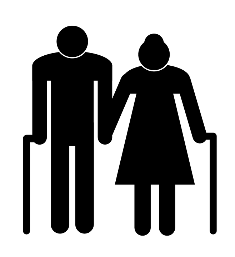

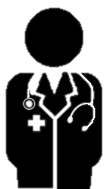

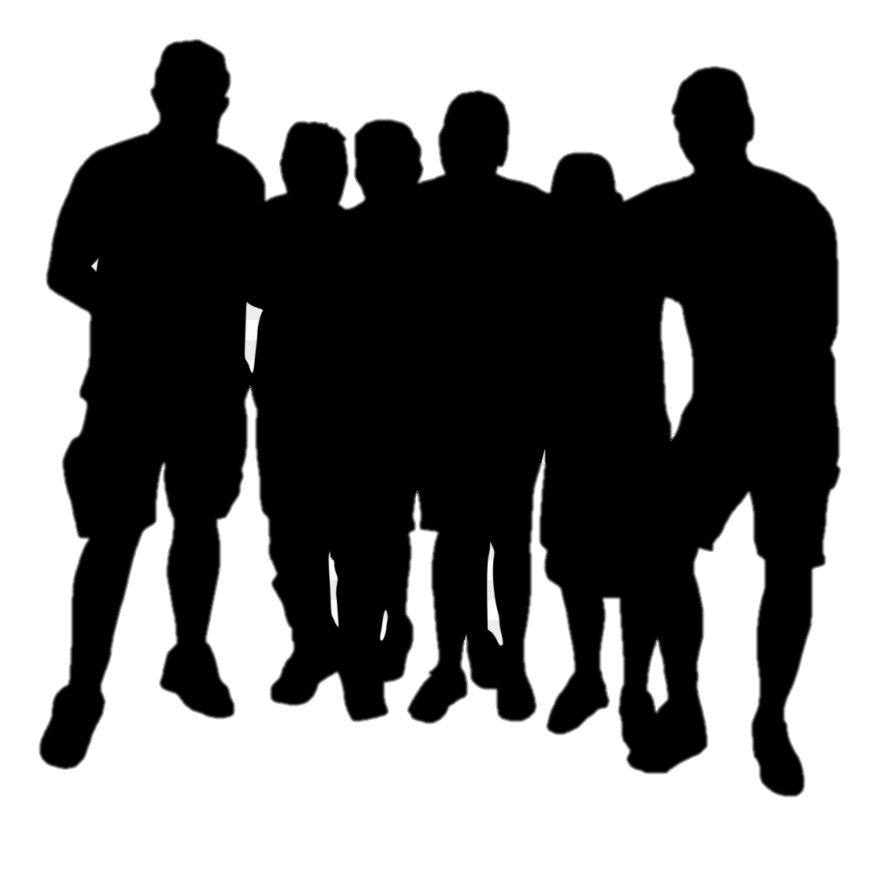


Husband

/Partner

Mother

Doctor

Grandparents

Friends/Families/Others

**Factor 4:** **Choice should be made using the advice of doctors, but the decision should be made together with the partner/husband (11 exemplars)**


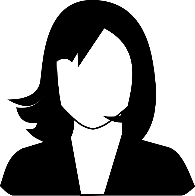

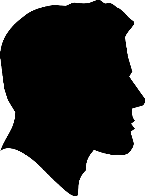

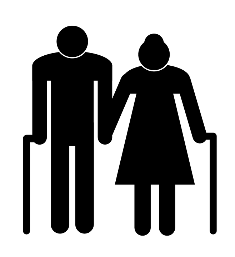

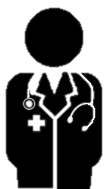

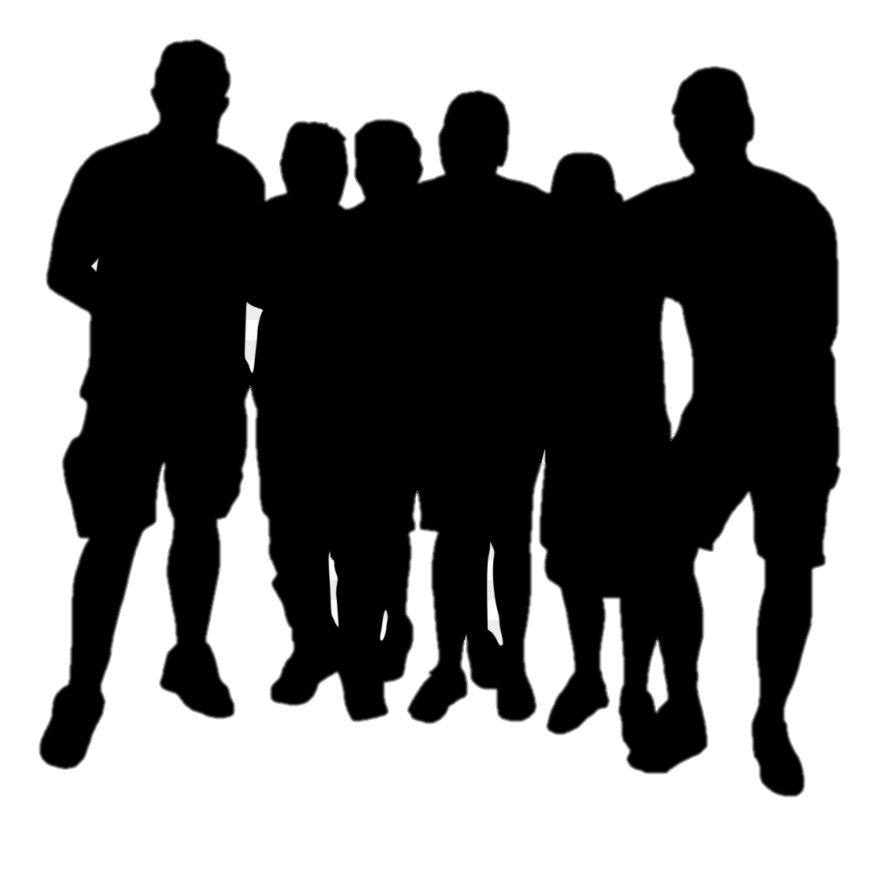


Husband

/Partner

Mother

Doctor

Friends/Families/Others

Grandparents

**Factor 5:** **Choice is a responsibility shared with the partner, family and doctors (9 exemplars).**


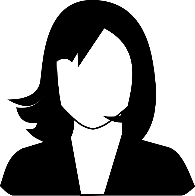

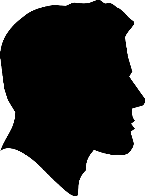

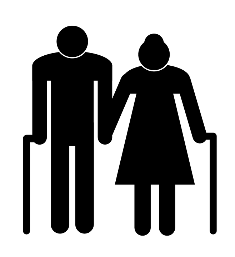

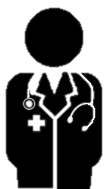

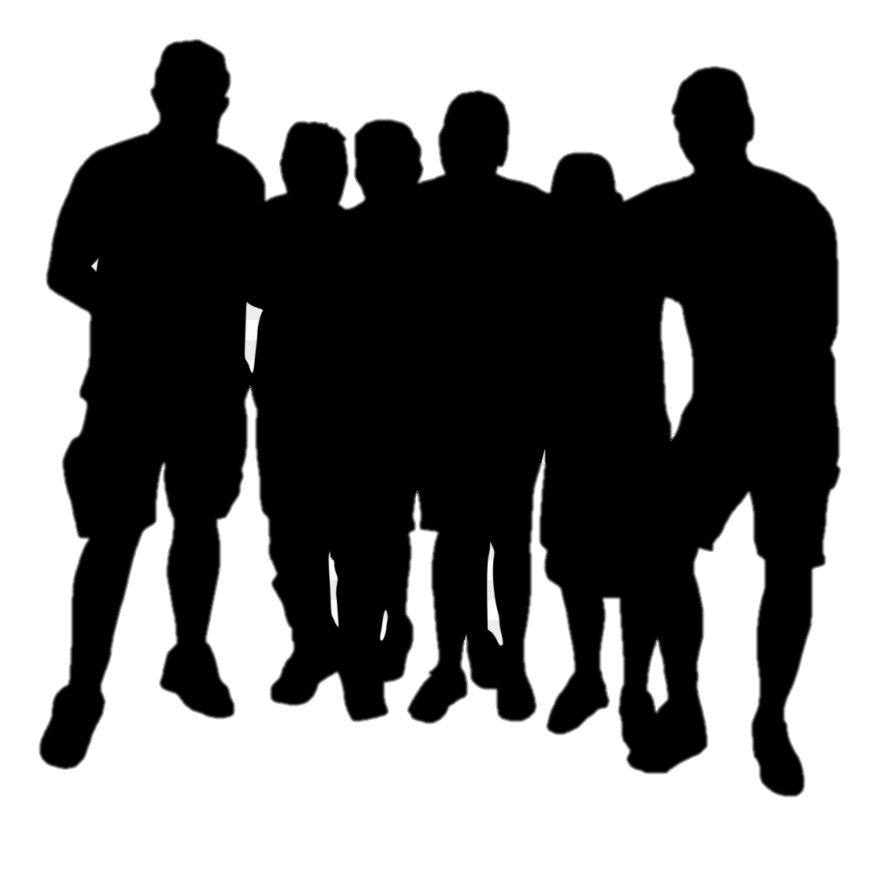


Husband

/Partner

Mother

Doctor

Friends/Families/Others

Grandparents
